# A Pair of New Antioxidant Phenolic Acid Stereoisomers Isolated from Danshen Injection (Lyophilized Powder)

**DOI:** 10.3390/molecules19021786

**Published:** 2014-02-04

**Authors:** Jing Liu, Jianfeng Zhao, Zhong Dai, Ruichao Lin, Gangli Wang, Shuangcheng Ma

**Affiliations:** National Institutes for Food and Drug Control, Beijing 100050, China

**Keywords:** Danshen Injection, phenolic acid, stereoisomer, antioxidant activity

## Abstract

A pair of new phenolic acid stereoisomers, (*R*)-norsalvianolic acid L (**1**) and (*S*)-norsalvianolic acid L (**2**), was isolated from the Danshen Injection (lyophilized powder). The structural elucidation and stereochemistry determination were achieved by spectroscopic and chemical methods including 1D, 2D NMR (^1^H-^1^H COSY, HSQC and HMBC) and circular dichroism experiments. Their antioxidant activities were assessed by the DPPH**^·^** and ABTS**^·+^** scavenging methods *in vitro* with microplate assay. The IC_50_ values of **1** were 6.9 and 9.7 μM respectively, which was close to the control salvianolic acid B (7.8 and 7.1 μM respectively), while the IC_50_ values of isomer **2** were 27.1 and 25.3 μM, respectively.

## 1. Introduction

In China *Salviae miltiorrhizae* Radix et Rhizoma, also called Danshen, is an important commonly used Traditional Chinese Medicine. It activates and promotes blood circulation, and is widely used for the treatment of coronary heart diseases, hepatitis and dysmenorrhea [1]. Its main effective constituents are known as the hydrophilic phenolic acids and lipophilic diterpenoids [2,3,4]. Recent investigations have focused on the mechanisms of action of its bioactive components [5,6] and development of new quality control methods [7,8].

Danshen Injection (lyophilized powder) is prepared by the process of water-extraction and ethanol precipitation from *S.* *miltiorrhizae* Radix et Rhizome. The injection is commonly used for treatment of cardiovascular diseases. Meanwhile, modern pharmacological studies demonstrate that one of the mechanisms of its cardioprotective action is the radical oxygen scavenging potential of the phenolic acids it contains [3,9], as many phenolic acids, such as rosmarinic acid, and salvianolic acid A and B which are isolated from the crude drug *S.* *miltiorrhizae* were reported to present strong antioxidant activities [10,11,12]. Meanwhile, assays using DPPH^·^ and ABTS^·+^ radicals are the widely-used spectrophotometric methods for determination of the antioxidant capacity due to the simple, rapid, sensitive and reproducible procedures [13,14].

**Figure 1:**
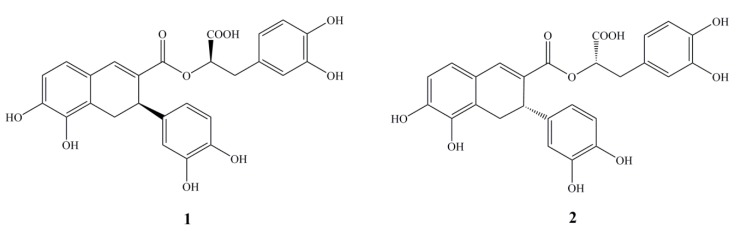
Structures of the stereoisomers isolated from Danshen Injection (lyophilized powder).

To elucidate the effective substances responsible for traditional function and indication, the phenolic constituents of Danshen Injection (lyophilized powder) were chemically investigated, and their radical scavenging capacities were measured by DPPH and ABTS assays. As a result, some phenolic acids such as danshensu and protocatechuic acid were isolated. The most interesting result, however, was the discovery of some new phenolic acid stereoisomers. In this paper, we report the isolation, structural identification and antioxidant activity of the stereoisomers (*R*)-norsalvianolic acid L (**1**) and (*S*)-norsalvianolic acid L (**2**) (Figure 1).

## 2. Results and Discussion

### 2.1. Structural Elucidation of the Isomers

Compound **1** was obtained as a yellow powder. It showed positive reaction with 1% FeCl_3_ test solution. The negative HRESIMS (*m/z* 493.1129 [M−H]^−^, calcd. for C_26_H_21_O_10_: 493.1140) gave the molecular formula of C_26_H_22_O_10_ with 16 degrees of unsaturation. The UV spectrum displayed absorptions at 221, 294 and 332 nm, in accordance with the reported UV absorptions of salvianolic acids A–C [15,16]. The IR spectrum indicated the presence of hydroxyl (3,368 cm^−1^), carbonyl (1,684 cm^−1^) and aromatic ring (1,612, 1,581, 1,521, 1,446 cm^−1^) functionalities in the structure of 1. The ^1^H-NMR spectrum of 1 presented nine downfield proton signals, including two sets of ABX coupling system protons [I: *δ* 6.86 (1H, d, 1.5, H-2'), *δ* 6.76 (1H, d, 8.0, H-5') and *δ* 6.66 (1H, dd, 7.5, 2.0, H-6'); II: *δ* 6.60 (1H, d, 2.0, H-12), *δ* 6.56 (1H, d, 8.0, H-15) and *δ* 6.44 (1H, dd, 8.5, 2.0, H-16)], one set of AB coupling system proton signals [*δ* 6.80 (1H, d, 8.0, H-5) and *δ* 6.73 (1H, d, 8.0, H-6)], together with one singlet aromatic proton signal at *δ* 7.68 (1H, s, H-4). There still existed two sets of AX_2_ coupling protons [I: *δ* 5.11 (1H, dd, 8.0, 4.5, H-8'), 3.09 (1H, dd, 14.0, 4.5, H-7'), 3.03 (1H, dd, 14.5, 8.0, H-7'); II: *δ* 4.01 (1H, d, 8.0, H-2), 3.38 (1H, br d, 16.5, H-1), 2.97 (1H, dd, 16.5, 8.5, H-1)]. The ^13^C-NMR spectrum exhibited 26 signals, of which two carbons were methylenes, 11 were methines and 13 were quaternary carbons, as shown in the DEPT spectrum. Further analysis demonstrated two carbonyl signals at *δ* 171.1 (COOH-9′) and *δ* 166.8 (COO-17); 11 quaternary aromatic carbons at *δ* 148.2 (C-7), 145.6 (C-3′), 145.2 (C-13), 144.7 (C-4′), 144.2 (C-14), 143.4 (C-8), 136.1 (C-11), 129.1 (C-1′), 128.5 (C-3), 126.1 (C-10) and 122.7 (C-9); 11 methine carbons at *δ* 138.4 (C-4), 122.1 (C-5), 121.8 (C-6′), 119.5 (C-16), 117.5 (C-2′), 116.0 (C-5′), 115.6 (C-15), 115.1 (C-12), 113.4 (C-6), 73.8 (C-8′) and 37.2 (C-2); as well as two methylene carbons at *δ* 37.5 (C-7′) and 30.4 (C-1), as shown in Table 1.

**Figure 2:**
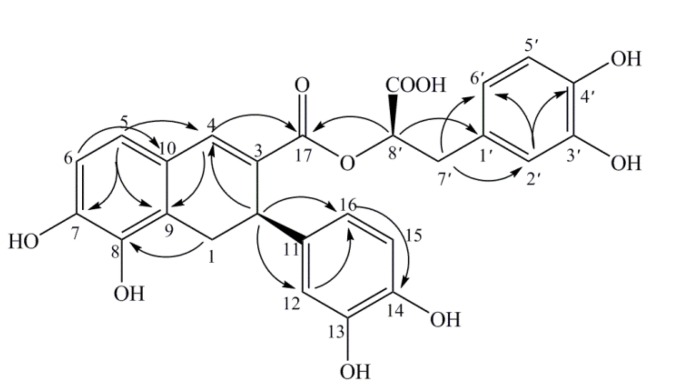
Key HMBC correlations (H→C) in compound 1.

The HMBC spectrum presented correlation signals from *δ* 7.68 (H-4) to 166.8 (COO-17)/136.1 (C-11)/128.5 (C-3)/126.1 (C-10)/122.7 (C-9)/37.2 (C-2), from *δ* 6.86 (H-2′) to δ 144.7 (C-4′)/121.8 (C-6′), from δ 6.80 (H-5) to δ 148.2 (C-7)/138.4 (C-4)/122.7 (C-9)/δ 143.4 (C-8), from δ 6.76 (H-5′) to δ 145.6 (C-3′)/δ 129.1 (C-1′), from δ 6.73 (H-6) to δ 148.2 (C-7)/143.4 (C-8)/126.1 (C-10), from δ 6.66 (H-6′) to δ 117.5 (C-2′), from δ 6.60 (H-12) to δ 144.2 (C-14)/119.5 (C-16)/115.7 (C-15), from δ 6.56 (H-15) to δ 145.2 (C-13)/136.1 (C-11), from δ 6.44 (H-16) to δ 144.2 (C-14)/115.1 (C-12). Additionally, there were also HMBC correlations from δ 5.11 (H-8′) to δ 171.1 (COOH-9′)/166.8 (COO-17)/129.1 (C-1′)/37.5 (C-7′), from δ 4.01 (H-2) to δ 138.4 (C-4)/136.1 (C-11)/128.5 (C-3)/122.7 (C-9)/119.5 (C-16)/115.1 (C-12), from δ 3.38/2.97(H-1) to δ 143.4 (C-8)/136.1 (C-11)/126.1 (C-10)/122.7 (C-9)/37.2 (C-2), as well as from δ 3.09/3.03 (H-7′) to δ 171.1 (COOH-9′)/129.1 (C-1′)/121.8 (C-6′)/117.5 (C-2′)/73.8 (C-8′) (Figure 2). Moreover, the ^1^H-^1^H COSY and HSQC spectra indicated the presence of such structural units as CH (H-8′)-CH_2_ (H-7′), CH (H-2)-CH_2_ (H-1), CH (H-5)=CH (H-6), CH (H-15) =CH (H-16) and CH (H-5′) =CH (H-6′). Based on the combined ^1^H-, ^13^C- NMR, HMBC, HSQC and ^1^H-^1^H COSY spectral data discussed above, the planar structure of compound 1 was elucidated as 3-(3,4-dihydroxyphenyl)-2-[3-(3,4-dihydroxyphenyl)-5, 6-dihydroxy-3, 4-dihydronaphthalene-2-carbonyloxy] propanoic acid. 

**Figure 3:**
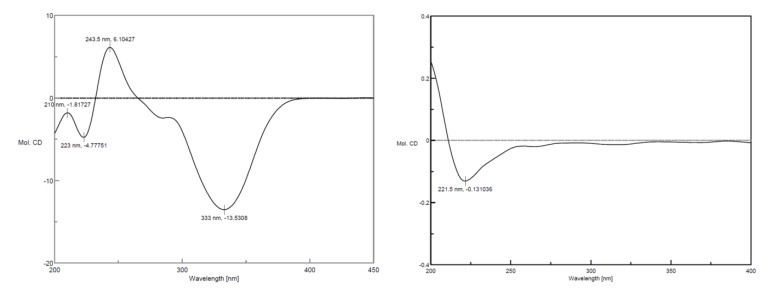
CD spectra of compound **1** and danshensu.

There were two chromophores containing л → л* electron transition systems, which made the application of exciton-coupled circular dichroism (ECCD) feasible to determine the absolute configuration of C2. The CD spectrum (Figure 3) presented Cotton effects at 333 nm (−) and 243.5 nm (+), which indicated the negative chirality and thus concluded the absolute configuration as 2(*R*) [17]. Meanwhile, the negative Cotton effect 223 nm (−) was basically identical with that of danshensu (Figure 3), so the absolute configuration of C8′ was determined as 8′(*R*) [18]. Therefore, the structure of compound **1** was assigned as (*R*)-3-(3, 4-dihydroxyphenyl)-2-[(*R*)-3-(3,4-dihydroxy-phenyl)-5,6-dihydroxy-3,4-dihydronaphthalene-2-carbonyloxy]propanoic acid (Figure 1), and it was named (*R*)-norsalvianolic acid L.

**Figure 4:**
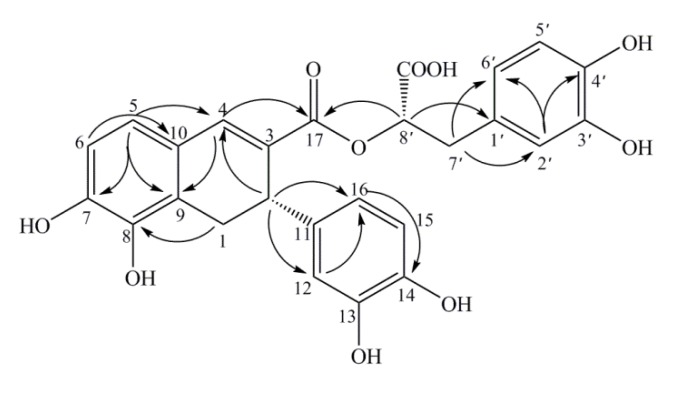
Key HMBC correlations (H→C) in compound **2**.

Compound **2**, isolated as a yellow powder, also possessed the molecular formula C_26_H_22_O_10_ according to its negative HRESIMS (*m/z* 493.1128 [M−H]^−^, calcd. for C_26_H_22_O_10_: 493.1140). The UV, IR and NMR data (Table 1) were almost identical to those for **1**, but both were obtained from the same fraction at different retention times. So compound **2** was deduced as an isomer of **1**, and the planar structure was further confirmed by the ^1^H-^1^H COSY, HSQC, and HMBC (Figure 4) spectra. 

**Figure 5:**
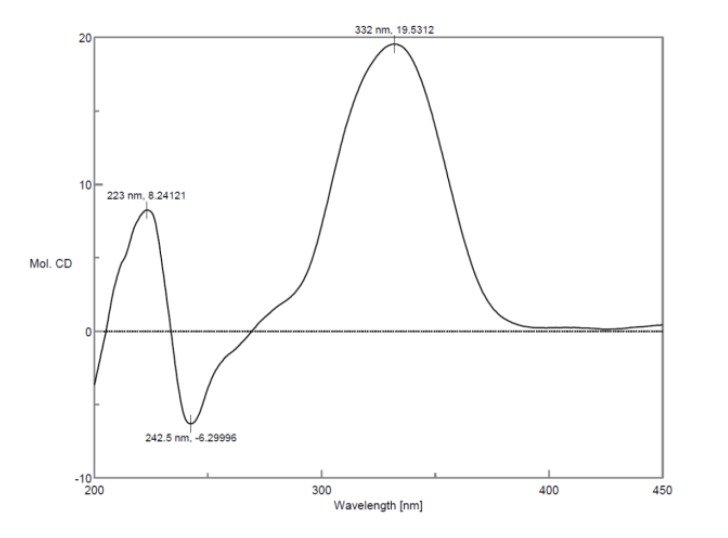
CD spectrum of compound **2**.

In addition, the specific rotation of compound **2** was [*α*]^20^_D_ +139.2° (*c* 0.38, CH_3_OH), opposite to that of compound **1**, [*α*]^20^_D_ −200° (*c* 0.21, CH_3_OH). Furthermore, the CD spectrum of **2** (Figure 5) presented Cotton effects at 332 nm (+), 242.5 nm (−), which indicated the isomeric characteristic and thus concluded the absolute configuration as 2(*S*), while the negative Cotton effect 223 nm (+) was on the contrary to that of compound **1**, so the absolute configuration of C8′ was determined as 8′(*S*). Thus, the structure of compound **2** was assigned as (*S*)-3-(3,4-dihydroxyphenyl)-2-[(*S*)-3-(3,4-dihydroxy-phenyl)-5, 6-dihydroxy-3, 4-dihydronaphthalene-2-carbonyloxy]propanoic acid (Figure 1), and it was named (*S*)-norsalvianolic acid L.

**Table 1:** NMR data of compounds **1** and **2** (acetone-d_6_; 500 MHz for ^1^H, 125 MHz for ^13^C).

position		1		2	
		*δ*_H_ (ppm)	*δ*_C_ (ppm)	*δ*_H_ (ppm)	*δ*_C_ (ppm)
1	CH_2_	3.38 (1H, br d, 16.5); 2.97 (1H, dd, 16.5, 8.5)	30.4	3.38 (1H, br d, 16.5); 2.97 (1H, dd, 16.5, 8.0)	30.5
2	CH	4.01 (1H, d, 8.0)	37.2	4.06 (1H, d, 8.0)	37.2
3	C		128.5		128.6
4	CH	7.68 (1H, s)	138.4	7.68 (1H, s)	138.7
5	CH	6.80 (1H, d, 8.0)	122.1	6.80 (1H, d, 8.5)	122.0
6	CH	6.73 (1H, d, 8.0)	113.4	6.72 (1H, d, 8.0)	113.4
7	C		148.2		148.2
8	C		143.4		143.4
9	C		122.7		122.6
10	C		126.1		126.2
11	C		136.1		135.9
12	CH	6.60 (1H, d, 2.0)	115.1	6.60 (1H, d, 2.0)	115.1
13	C		145.2		145.2
14	C		144.2		144.3
15	CH	6.56 (1H, d, 8.0)	115.6	6.58 (1H, d, 8.0)	115.7
16	CH	6.44 (1H, dd, 8.5, 2.0)	119.5	6.48 (1H, dd, 9.0, 1.5)	119.6
17	C		166.8		167.0
1′	C		129.1		129.2
2′	CH	6.86 (1H, d, 1.5)	117.5	6.79 (1H, br s)	117.3
3′	C		145.6		145.5
4′	C		144.7		144.7
5′	CH	6.76 (1H, d, 8.0)	116.0	6.69 (1H, d, 8.0)	116.0
6′	CH	6.66 (1H, dd, 7.5, 2.0)	121.8	6.50 (1H, dd, 9.5, 1.5)	121.9
7′	CH_2_	3.09 (1H, dd, 14.0, 4.5); 3.03 (1H, dd, 14.5, 8.0)	37.5	3.04 (1H, dd, 14.0, 4.5); 2.98 (1H, dd, 13.5, 8.0)	37.6
8′	CH	5.11 (1H, dd, 8.0, 4.5)	73.8	5.14 (1H, dd, 8.0, 4.5)	73.9
9′	C		171.1		171.1

### 2.2. *In Vitro* Antioxidant Activity

Both the DPPH (1,1-diphenyl-2-picrylhydrazyl) and ABTS (2,2′-azino-bis-3-ethylbenzthiazoline-6-sulphonic acid) assays are commonly used radical scavenging assays to detect antioxidant capacity. These two methods are simple, rapid, sensitive and reproducible [13,14,19]. In our study, the antioxidant activities of the stereoisomers were characterized by DPPH and ABTS assays. According to the respective calculations described in Section 3.3.1 and Section 3.3.2, it was shown that the IC_50_ values of compound **1** were 6.9 μM (DPPH) and 9.7 μM (ABTS), respectively. The result was close to the control salvianolic acid B (7.8 μM and 7.1 μM respectively). Meanwhile, the IC_50_ values of the isomer **2** were 27.1 and 25.3 μM, respectively (Table 2). The results indicated that both isomers possessed potential antioxidant activities and the stereo structure is important for the activity. 

**Table 2:** DPPH^·^ and ABTS^·+^ scavenging activity (*in vitro*).

	DPPH^·^ scavenging activity				ABTS^·+^ scavenging activity			
**Compounds**	**DS**	**1**	**2**	**Sal B**	**DS**	**1**	**2**	**Sal B**
**IC_50_ (μM)**	9.5	6.9	27.1	7.8	11.1	9.7	25.3	7.1

DS represents the Danshen Injection (lyophilized powder), from which the compounds were isolated.

## 3. Experimental

### 3.1. General

Optical rotations were determined on an AUTOPOL IV Automatic Polarimeter (Rudolph, Hackettstown, NJ, USA). IR spectra were recorded on a Nicolet 5700 FT-IR spectrometer (Thermo Scientific, Waltham, MA, USA) by a microscope transmission method. UV spectra were obtained on an Agilent 1200 series UV spectrometer (Agilent, Santa Clara, CA, USA). 1D NMR and 2D NMR experiments were performed on Inova 500 spectrometers (Varian, Santa Clara, CA, USA). Chemical shifts are given in δ (ppm) with solvent (acetone-*d*_6_) and with TMS as internal standard. ESIMS were measured on Agilent 6320 Ion Trap mass spectrometer. HRESIMS were measured on an Agilent 6210 TOF mass spectrometer. CD spectra were measured on a JASCO J-815 spectropolarimeter (JASCO, Easton, MD, USA) with a 0.1 cm cell in methanol at room temperature at the following conditions: speed 200 nm/min, time constant 1 s, bandwidth 2.0 nm. Macroporous resin AB-8 (50 mesh, Tianjin Nankai Chemistry Company, Tianjin, China), polyamide (30–60 mesh, Jiangsu Linjiang Chemical Reagents Factory, Taixing, China), Sephadex LH-20 (Amersham Pharmacia Biotech AB, Uppsala, Sweden) and ODS (45–70 µm, Fuji Silysia, Kasugai, Japan) were used for column chromatography. Silica gel GF_254_ plates for TLC were purchased from Qingdao Marine Chemical Company, Qingdao, China. HPLC was carried out on an Elite P230 instrument equipped with a UV 230^+^ detector (Elite Analytical, Dalian, China), employing a preparative column (Phenomenex Luna Su C18 (2) 21.2 × 250 mm, 5 μm) and an analytical column (Macherey-Nagel Nucleoqur Nucleodur C_18_ Pyramid, 4.6 × 250 mm, 5 µm). In the antioxidant assay, a SpectraMax 190 Absorbance Microplate Reader (Molecular Devices, Sunnyvale, CA, USA) and 96 Well Cell Culture Cluster (Costar, Corning, NY, USA) were used. 1,1-Diphenyl-2-picrylhydrazyl (DPPH) and 2, 2′-azino-bis(3-ethylbenzthiazoline-6-sulfonic acid) (ABTS) were purchased from Sigma (Sigma-Aldrich GmbH, Stenheim, Germany). Solvents of analytical grade were purchased from Beijing Chemical Company, Beijing, China, while those of spectroscopy grade were from J. T. Baker (Center Valley, PA, USA). Danshen Injection (lyophilized powder) was provided by Second Chinese Medicine Factory, Harbin Pharm. Group Co. Ltd. (Haerbin, China). Reference standards of sodium danshensu and salvianolic acid B was from National Institutes for Food and Drug Control (Beijing, China). 

### 3.2. Compound Isolation

The Danshen Injection (lyophilized powder) (120.7 g) was firstly dissolved in water, and then subjected to AB-8 macroporous resin column chromatography eluted by a gradient of EtOH–H_2_O (0:100, 30:70, 60:40 and 95:5) to afford four fractions (Fr. A–D). Fr. B (26.1 g) was subsequently chromatographed over a polyamide column using a gradient of EtOH-H_2_O (0:100, 30:70, 60:40 and 95:5) as eluent to give four fractions (Fr. B1–B4). Fr. B3 (1.70 g) was purified by Sephadex LH-20 and ODS columns. Finally, compounds **1** (33 mg) and **2** (7 mg) were obtained by preparative HPLC with CH_3_CN-0.05%CF_3_COOH (24:76, v/v) as eluting mobile phase.

### 3.3. Antioxidant Activity *In Vitro*

#### 3.3.1. DPPH Assay

During the experiment, a microplate reader and 96-well plates were used to carry out the determination of the spectrum absorption values [13,19]. In the method, DPPH^·^ methanol solution (50 μL, 100 μg/mL) was added to 200 μL samples of different concentration (3.1‒100 μg/mL) of the test compounds. These solutions were gently mixed and incubated in dark for 30 min at room temperature. The absorbances of the resulting solutions were measured at 517 nm. For preparation of the standard curve, different concentrations of DPPH^·^ methanol solutions (5–50 μg/mL) were used. The DPPH^·^ concentration (μg/mL) in the reaction medium was calculated from the following calibration curve, determined by linear regression (*r*^2^: 0.9985):

Abs (λ_517_) = 0.022 × [DPPH^·^] + 0.0145

The scavenging capability of tested compounds was calculated using the following equation:

DPPH^·^ scavenging activity (%) = 1 − λ_517-S_/λ_517-C_

λ_517-C_ is absorbance of a control with no radical scavenger and λ_517-S_ is absorbance of the remaining DPPH^·^ in the presence of scavenger.

#### 3.3.2. ABTS Assay

The ABTS^·+^ radical were performed by reacting ABTS (1.1 mg/mL) and potassium persulfate (K2S2O8, 0.68 mg/mL), and then storing in the dark for 6 h at room temperature [14,19]. Then ABTS^·+^ (50 μL) was added to 200 μL samples of different concentrations (3.1–100 μg/mL). These solutions were gently mixed and incubated in dark for 30 min at room temperature. Then absorbances of the resulting solutions were measured at 734 nm. Different concentrations of ABTS^·+^ methanol solutions (55–220 μg/mL) were used to prepare the standard curve. The ABTS^·+^ concentration (μg/mL) in the reaction medium was calculated from the following calibration curve, determined by linear regression (*r*^2^: 0.9985):

Abs (λ_734_) = 0.0118 × [ABTS^·+^] + 0.109

The scavenging capability of tested compounds was calculated using the following equation:

ABTS^·+^ scavenging activity (%) = 1 − λ_734-S_/λ_734-C_

where λ_734-C_ is absorbance of a control with no radical scavenger and λ_734-S_ is absorbance of the remaining ABTS^·+^ in the presence of scavenger.

## 4. Conclusions

The bioactive constituents of Danshen Injection (lyophilized powder) were chemically studied and a new pair of isomers was isolated. Most of their spectral data were the same or similar to each other, except for the specific rotation and circular dichroism spectra. In our study, their radical scavenging capacities were measured by DPPH and ABTS assays. According to the results, both stereoisomers possessed potential antioxidant activity and that of compound 1 was closer to that of the control salvianolic acid B. Also it is interesting to find that the stereo structures do have effects on the antioxidant activities. 

## Supplementary Materials

Supplementary materials can be accessed at: http://www.mdpi.com/1420-3049/19/2/1786/s1.
