# Supplementary material for: A Pair of New Antioxidant Phenolic Acid Stereoisomers Isolated from Danshen Injection (Lyophilized Powder)
Source: Molecules. 2014 Feb 4;19(2):1786–94. doi: 10.3390/molecules19021786 (PMC6271289; doi:10.3390/molecules19021786)
Supplement: Supplementary file 1 [file molecules-19-01786-s001.pdf]

Supplementary Materials

Contents

MS, UV, IR and NMR (1D and 2D) spectra of compound **1** S1

MS, UV, IR and NMR (1D and 2D) spectra of compound **2** S11

HPLC-UV-ESI-MS^n chromatograms of Danshen Injection (lyophilized powder) S21

Figure S1. ESI-MS spectrum of compound **1**.

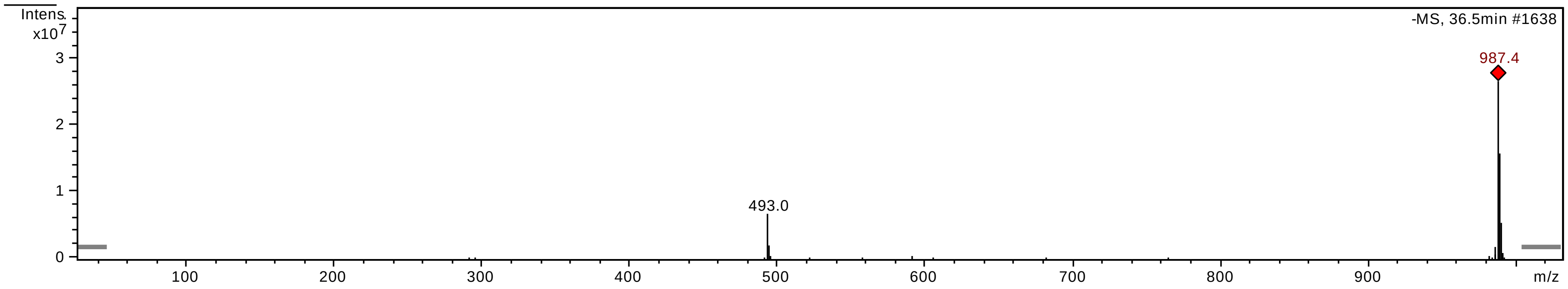

Figure S2. HR ESI-MS spectrum of compound **1**.

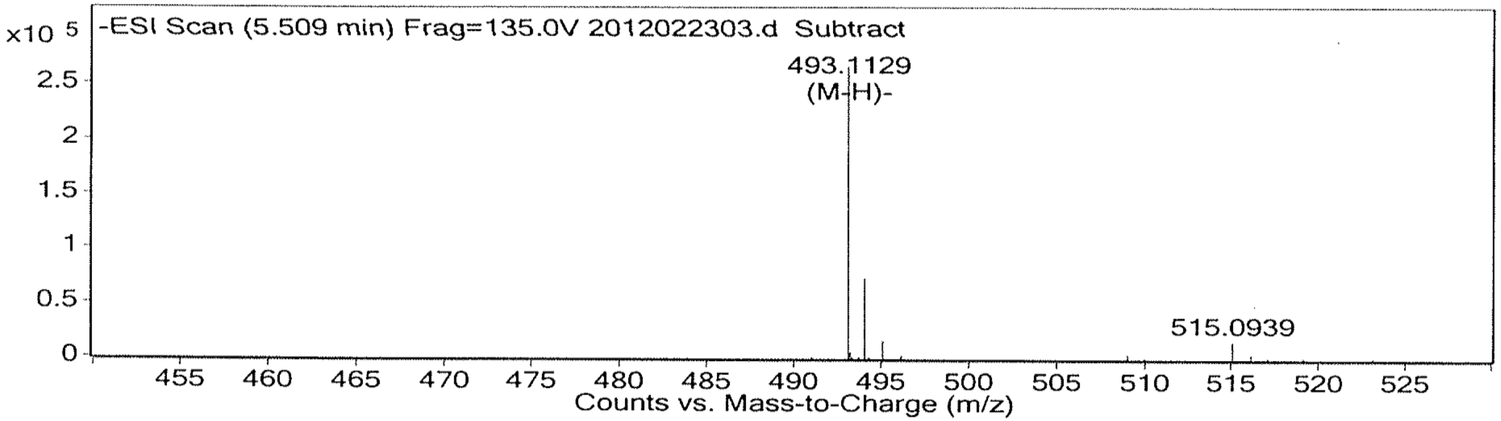

MS Formula Results: - Scan (5.509 min) Sub (2012022303.d)

m/z	Ion	Formula	Abundance
493.1129	(M-H)-	C26 H21 O10	265700.4

Best	Formula (M)	Ion Formula	Calc m/z	Score	Cross S	Mass	Calc Mass	Diff (ppm)	Abs Diff (ppm)	Abund Match	Spacing Mat	Mass Match	m/z	DBE
☑	C26 H22 O10	C26 H21 O10	493.114	99.89		494.1202	494.1213	2.23	2.23	99.92	99.95	99.83	493.1129	16
☐	C27 H18 N4 O6	C27 H17 N4 O6	493.1154	99.34		494.1202	494.1226	4.93	4.93	99.07	99.99	99.17	493.1129	21
☐	C21 H22 N2 O12	C21 H21 N2 O12	493.11	99.11		494.1202	494.1173	-5.92	5.92	98.9	99.97	98.8	493.1129	12
☐	C30 H22 O5 S	C30 H21 O5 S	493.1115	98.71		494.1202	494.1188	-2.84	2.84	96.2	99.72	99.72	493.1129	20
☐	C31 H18 N4 O S	C31 H17 N4 O S	493.1129	98.43		494.1202	494.1201	-0.14	0.14	94.75	99.72	100	493.1129	25
☐	C18 H26 N2 O12 S	C18 H25 N2 O12 S	493.1134	98.14		494.1202	494.1206	0.9	0.9	93.94	99.52	99.97	493.1129	7
☐	C23 H26 O10 S	C23 H25 O10 S	493.1174	97.82		494.1202	494.1247	9.05	9.05	97.34	99.6	97.23	493.1129	11
☐	C27 H26 O5 S2	C27 H25 O5 S2	493.1149	97.36		494.1202	494.1222	3.98	3.98	92.22	99.36	99.46	493.1129	15
☐	C22 H26 N2 O7 S2	C22 H25 N2 O7 S2	493.1109	97.21		494.1202	494.1181	-4.17	4.17	91.87	99.24	99.4	493.1129	11
☐	C33 H18 O5	C33 H17 O5	493.1081	97.14		494.1202	494.1154	-9.65	9.65	95.28	99.95	96.85	493.1129	25
☐	C34 H14 N4 O	C34 H13 N4 O	493.1095	97.06		494.1202	494.1168	-6.96	6.96	92.46	99.98	98.35	493.1129	30
☐	C28 H22 N4 O S2	C28 H21 N4 O S2	493.1162	96.91		494.1202	494.1235	6.67	6.67	92.24	99.35	98.48	493.1129	20
☐	C14 H26 N2 O17	C14 H25 N2 O17	493.1159	96.22		494.1202	494.1231	5.97	5.97	88.81	99.96	98.78	493.1129	3
☐	C34 H22 S2	C34 H21 S2	493.109	95.54		494.1202	494.1163	-7.91	7.91	88.33	99.5	97.87	493.1129	24
☐	C13 H26 N4 O14 S	C13 H25 N4 O14 S	493.1093	95.17		494.1202	494.1166	-7.26	7.26	86.55	99.44	98.21	493.1129	3

Figure S3. UV spectrum of compound **1**.

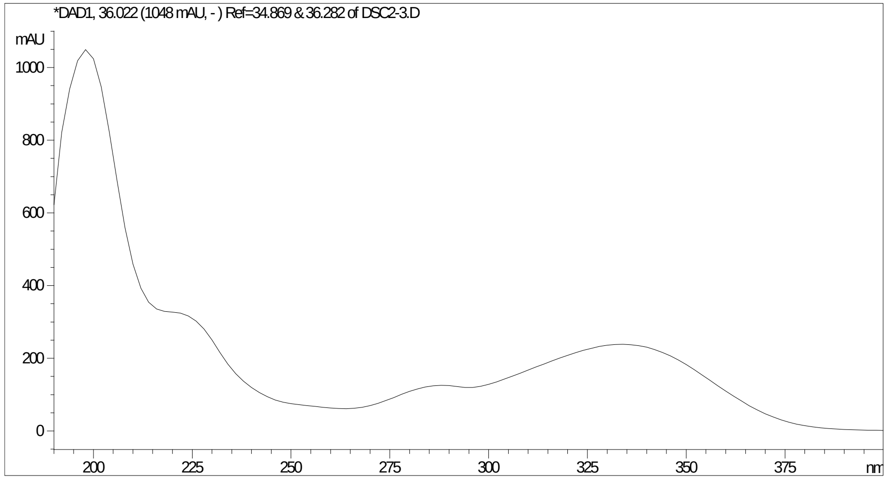

Figure S4. IR spectrum of compound **1**.

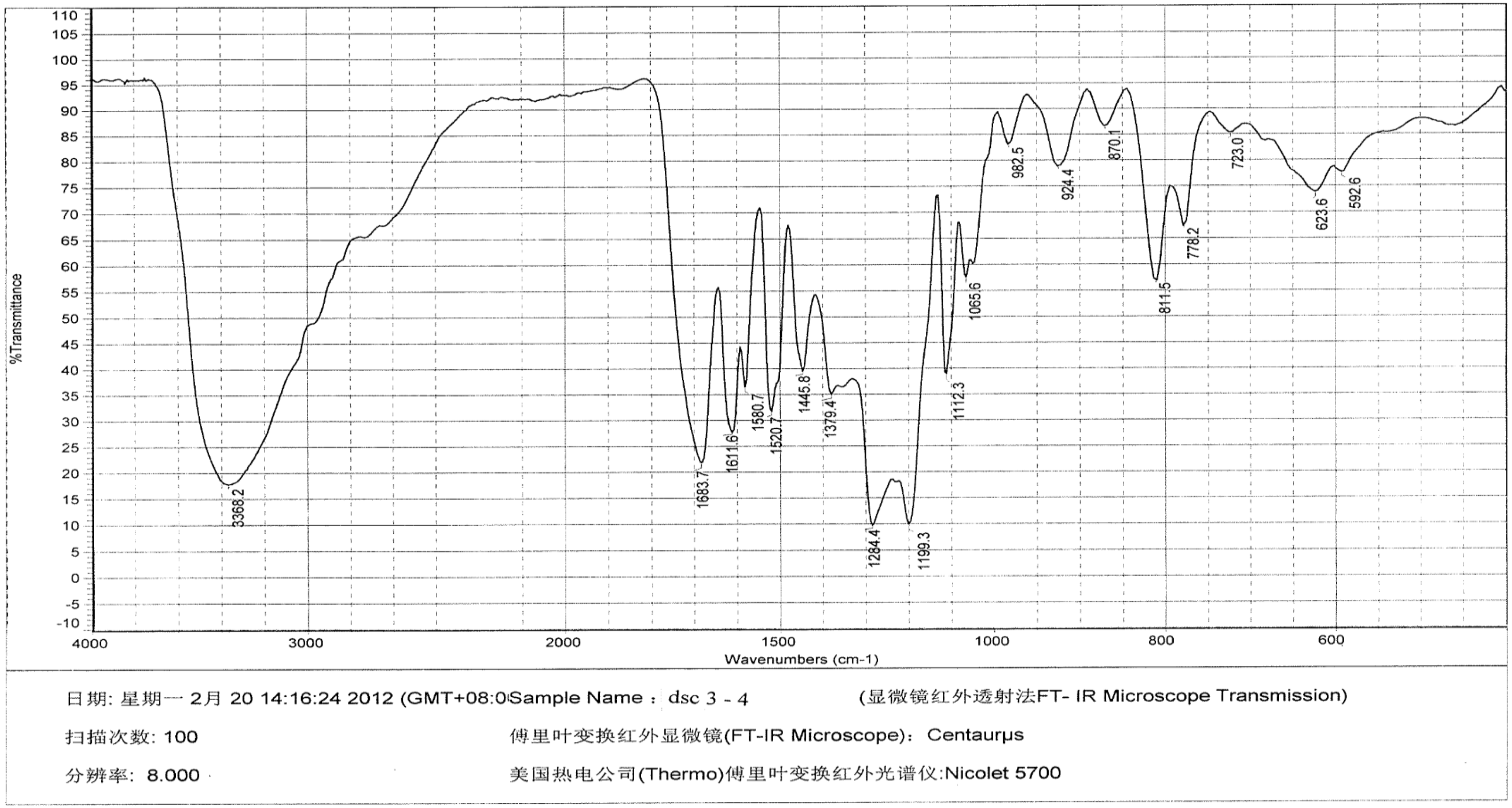

Date: Monday, Feb 20th, 14:16:24 2012 (GMT+08:0Sample Name: dsc 3-4) (Microscopy infrared transmission method FT-IR Microscope Transmission)
Numbers of scan: 100 Fourier transform infrared spectroscopy (FT-IR Microscope): Centaurus
Resolution ratio: 8,000 America Thermo Electron Corporation (Thermo) Fourier transform infrared spectrometer: Nicolet 5700.

Figure S5. ^{1}H NMR spectrum (acetone-d_6, 500 MHz) of compound **1**.

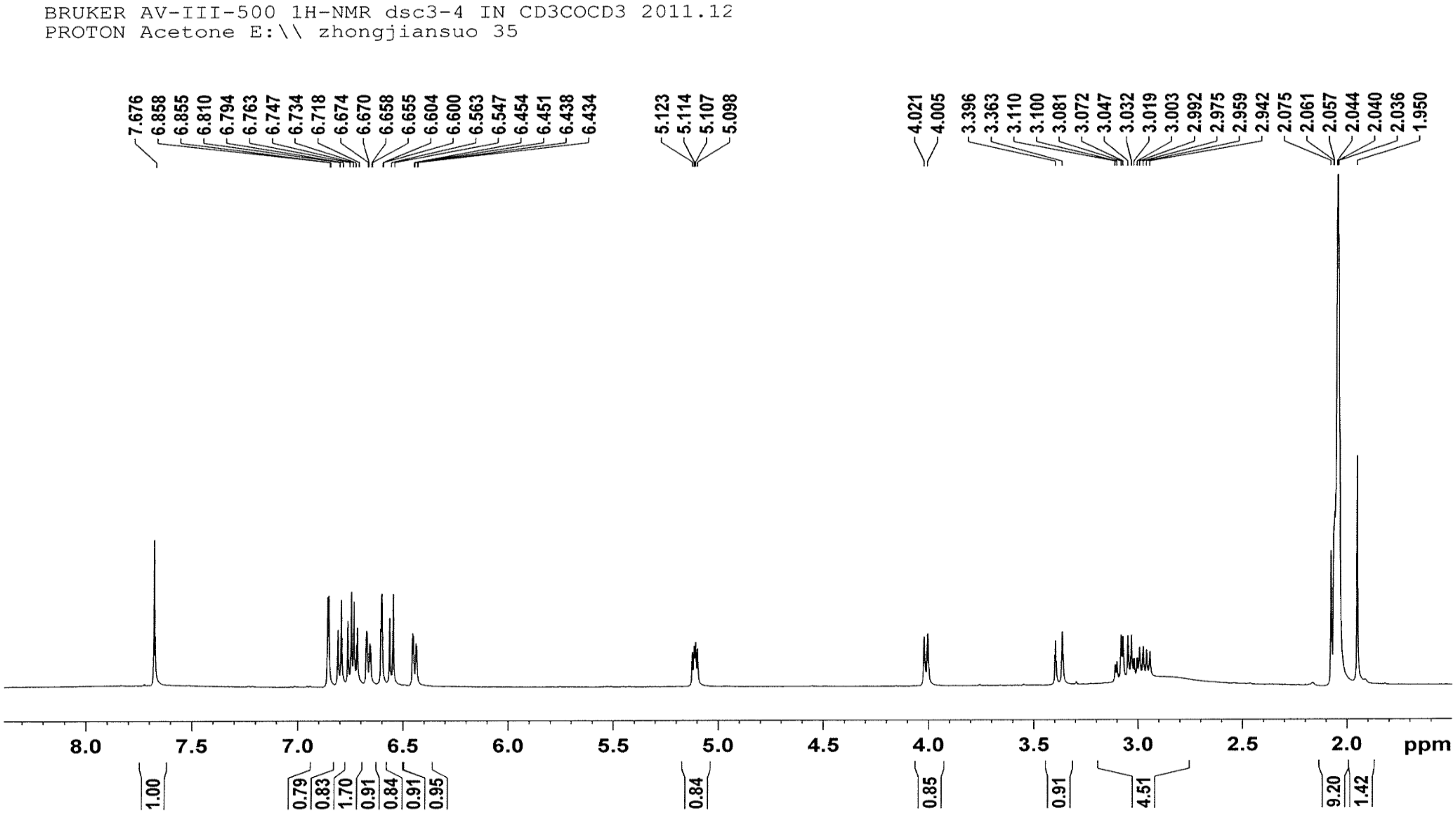

Figure S6. ^{13}C NMR spectrum (acetone-d_6, 125 MHz) of compound **1**.

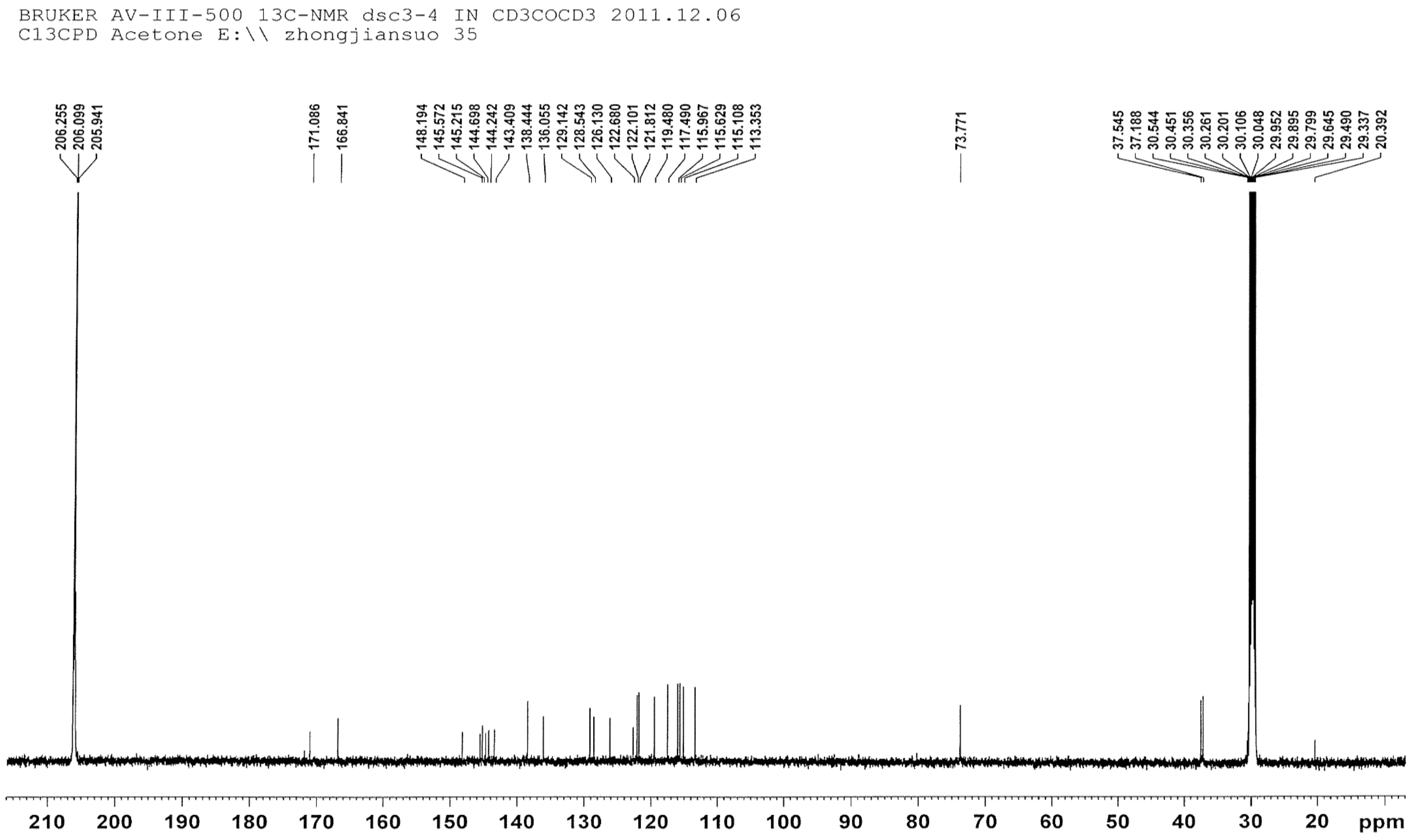

Figure S7. DEPT spectrum of compound **1**.

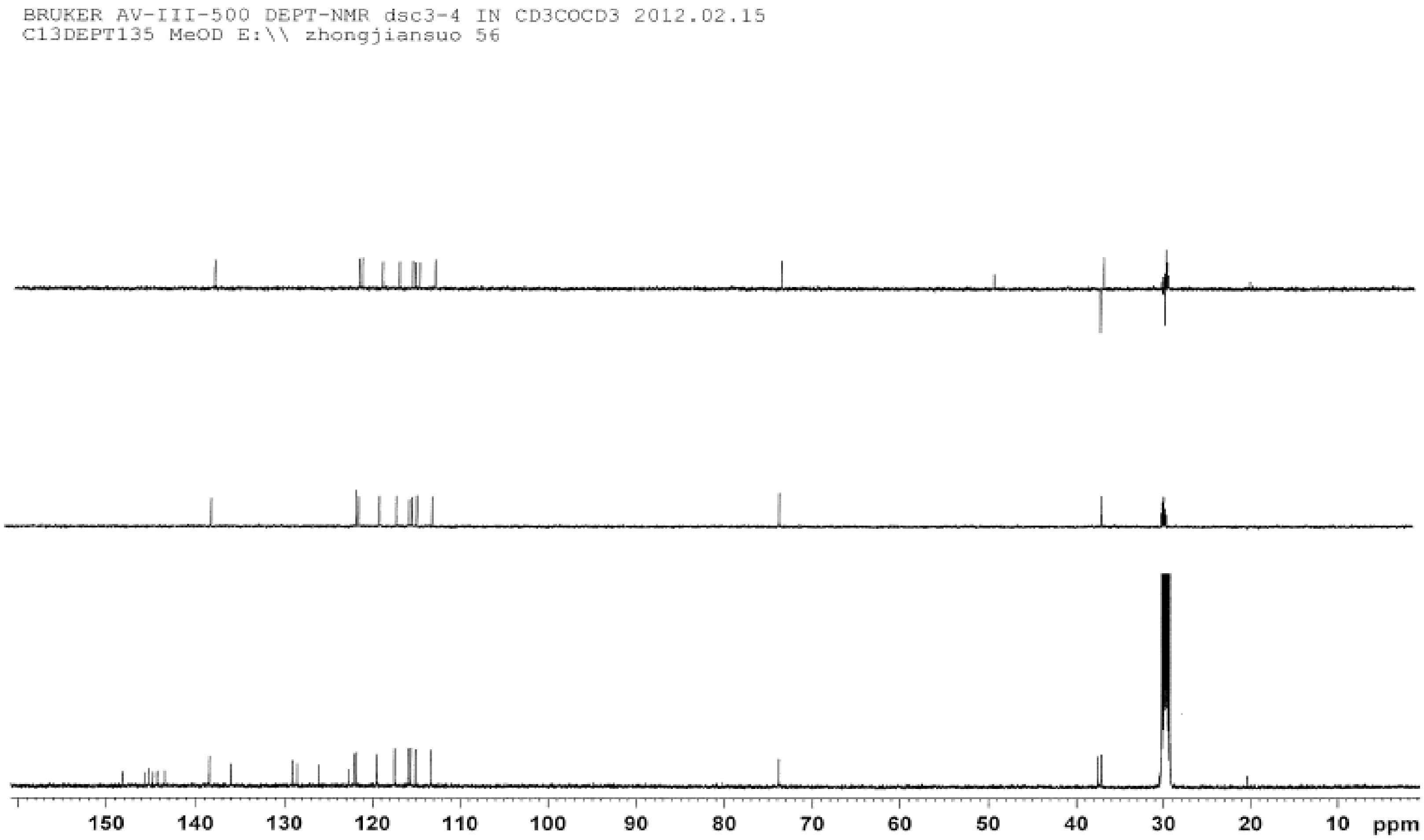

Figure S8. HSQC spectrum of compound **1**.

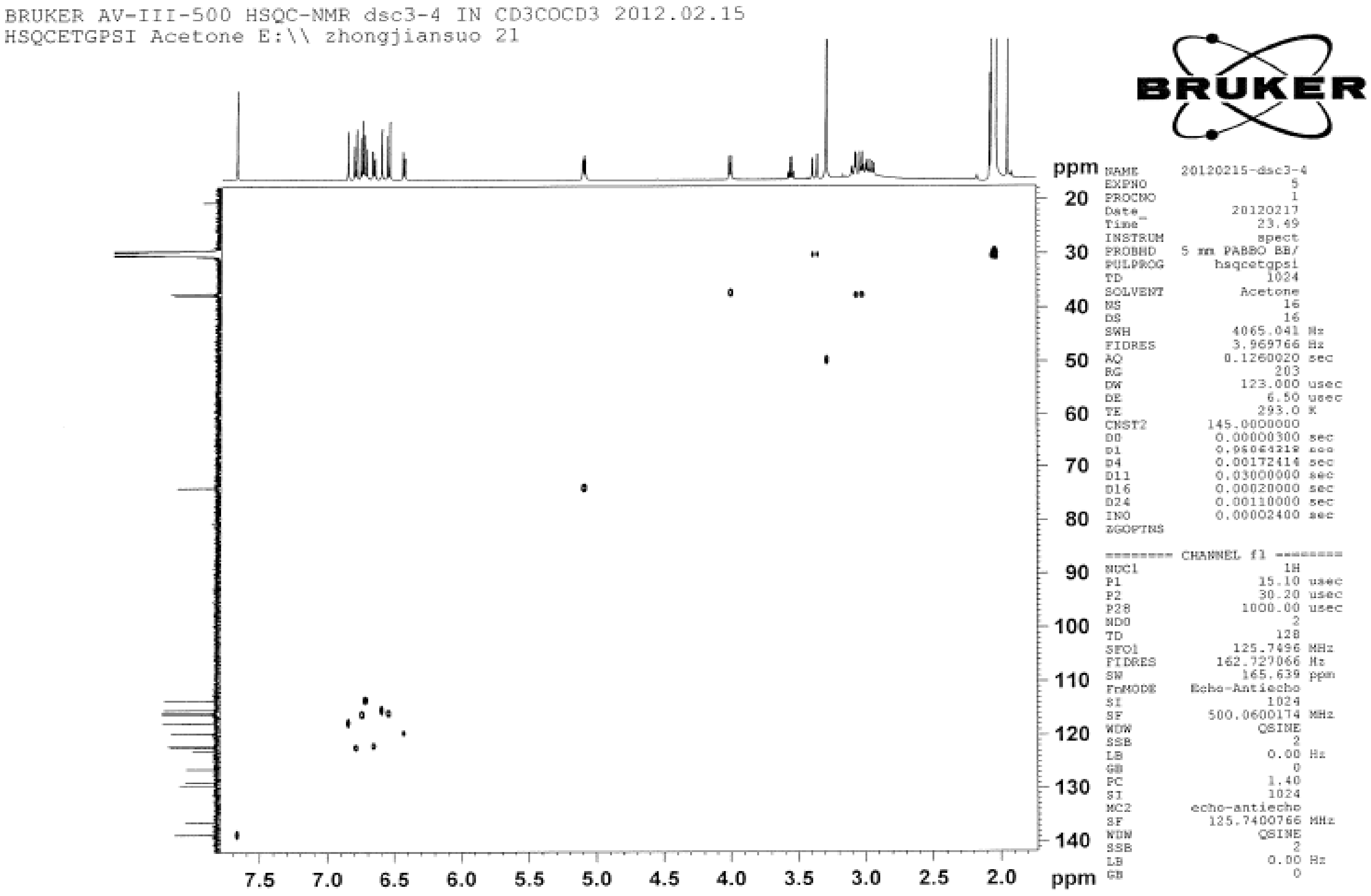

Figure S9. HMBC spectrum of compound **1**.

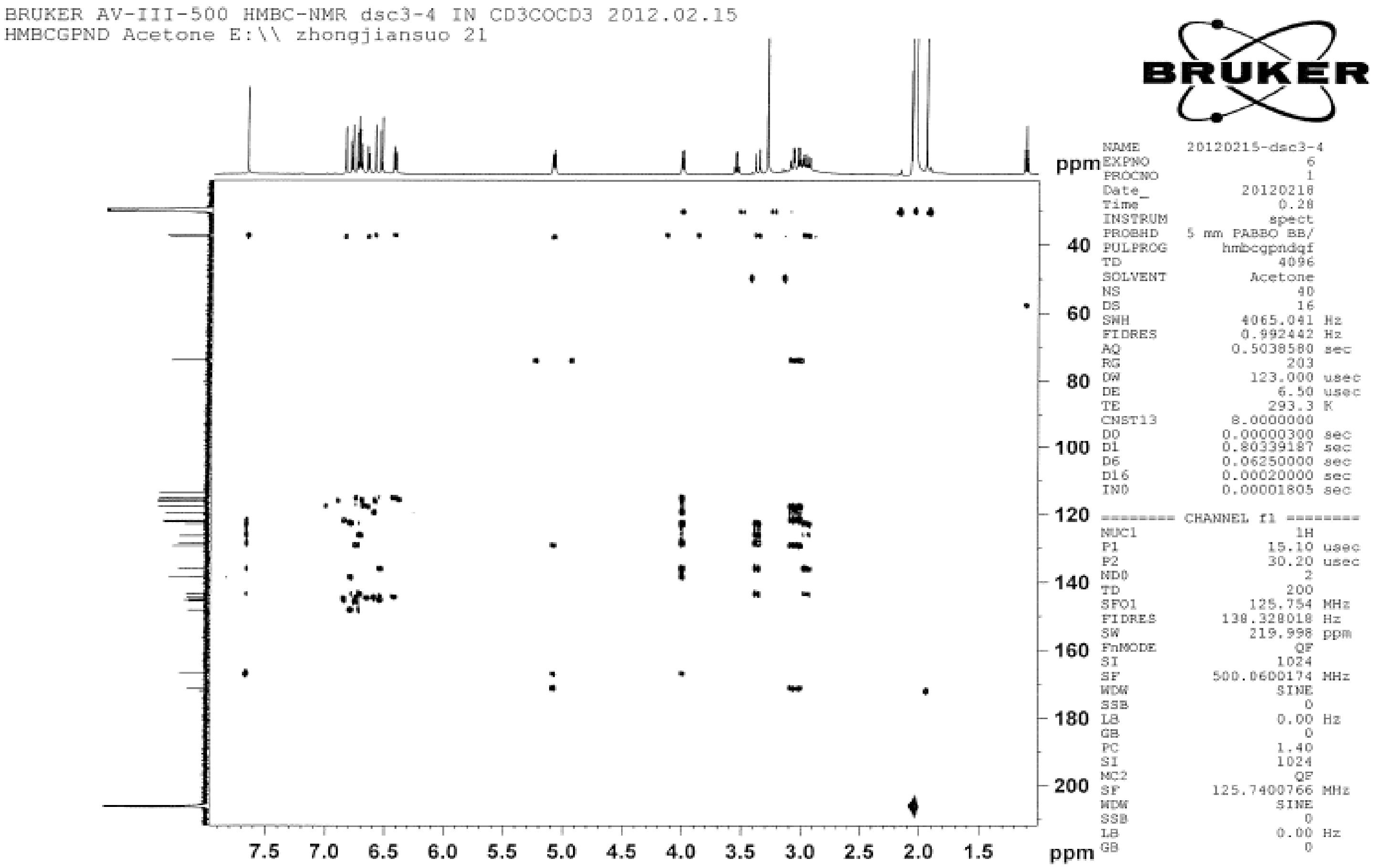

Figure S10. ^1H-^1H COSY spectrum of compound **1**.

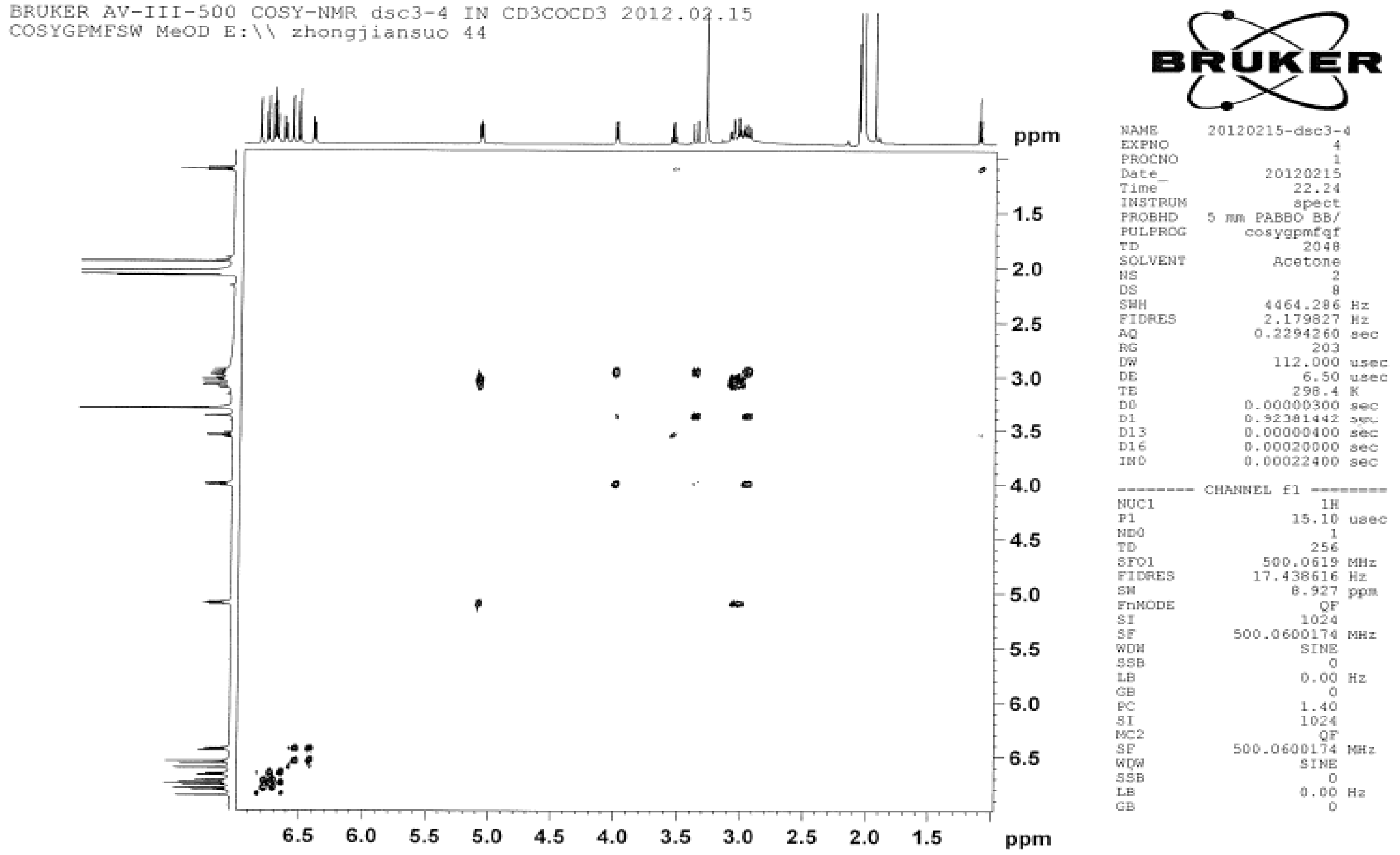

Figure S11. ESI-MS spectrum of compound **2**.

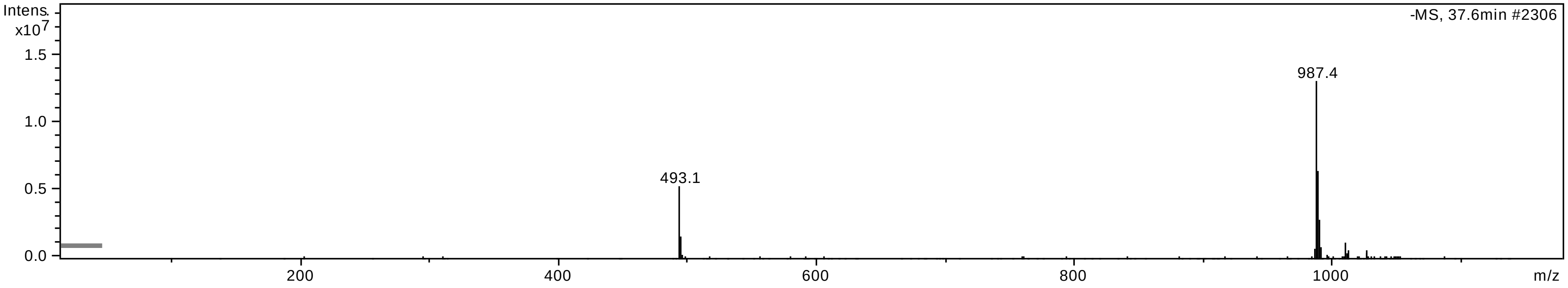

Figure S12. HR ESI-MS spectrum of compound **2**.

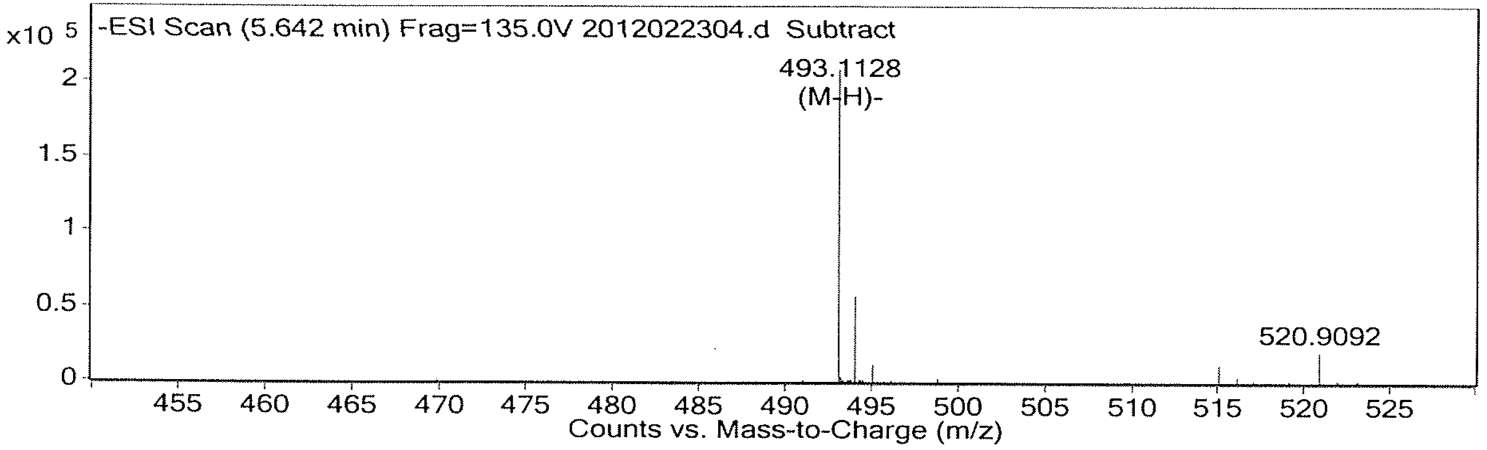

MS Formula Results: - Scan (5.642 min) Sub (2012022304.d)

m/z	Ion	Formula	Abundance
493.1128	(M-H)-	C26 H21 O10	207723.4

Best	Formula (M)	Ion Formula	Calc m/z	Score	Cross S	Mass	Calc Mass	Diff (ppm)	Abs Diff (ppm)	Abund Match	Spacing Mat	Mass Match	m/z	DBE
✓	C26 H22 O10	C26 H21 O10	493.114	99.86		494.12	494.1213	2.56	2.56	99.89	99.98	99.77	493.1128	16
	C27 H18 N4 O6	C27 H17 N4 O6	493.1154	99.29		494.12	494.1226	5.26	5.26	99.11	99.99	99.05	493.1128	21
	C21 H22 N2 O12	C21 H21 N2 O12	493.11	99.16		494.12	494.1173	-5.59	5.59	98.85	100	98.93	493.1128	12
	C30 H22 O5 S	C30 H21 O5 S	493.1115	98.62		494.12	494.1188	-2.51	2.51	95.65	99.86	99.78	493.1128	20
	C31 H18 N4 O S	C31 H17 N4 O S	493.1129	98.33		494.12	494.1201	0.19	0.19	94.27	99.85	100	493.1128	25
	C18 H26 N2 O12 S	C18 H25 N2 O12 S	493.1134	97.99		494.12	494.1206	1.23	1.23	93.3	99.71	99.95	493.1128	7
	C23 H26 O10 S	C23 H25 O10 S	493.1174	97.59		494.12	494.1247	9.38	9.38	96.72	99.78	97.02	493.1128	11
	C33 H18 O5	C33 H17 O5	493.1081	97.24		494.12	494.1154	-9.32	9.32	95.26	99.97	97.06	493.1128	25
	C34 H14 N4 O	C34 H13 N4 O	493.1095	97.14		494.12	494.1168	-6.63	6.63	92.49	99.98	98.5	493.1128	30
	C27 H26 O5 S2	C27 H25 O5 S2	493.1149	97.09		494.12	494.1222	4.31	4.31	91.21	99.6	99.36	493.1128	15
	C22 H26 N2 O7 S2	C22 H25 N2 O7 S2	493.1109	97.02		494.12	494.1181	-3.84	3.84	90.84	99.5	99.49	493.1128	11
	C28 H22 N4 O S2	C28 H21 N4 O S2	493.1162	96.62		494.12	494.1235	7.01	7.01	91.3	99.58	98.33	493.1128	20
	C14 H26 N2 O17	C14 H25 N2 O17	493.1159	96.11		494.12	494.1231	6.3	6.3	88.64	100	98.65	493.1128	3
	C34 H22 S2	C34 H21 S2	493.109	95.4		494.12	494.1163	-7.57	7.57	87.4	99.71	98.05	493.1128	24
	C13 H26 N4 O14 S	C13 H25 N4 O14 S	493.1093	95.1		494.12	494.1166	-6.93	6.93	85.89	99.62	98.36	493.1128	3

Figure S13. UV spectrum of compound **2**.

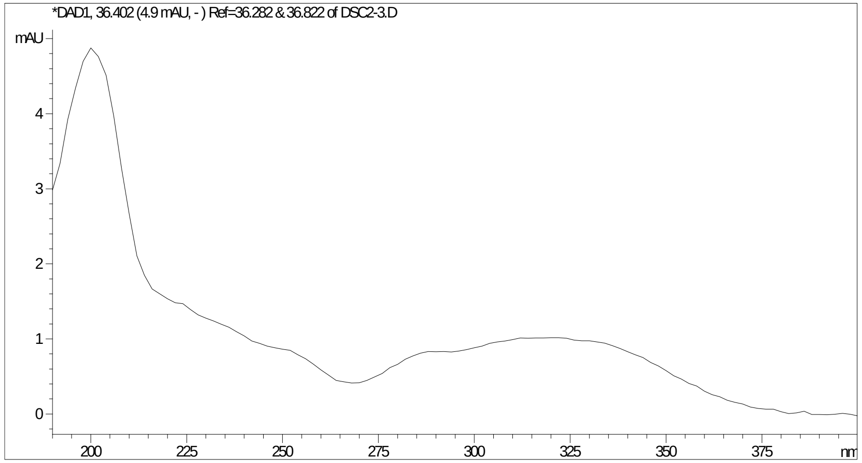

Figure S14. IR spectrum of compound **2**.

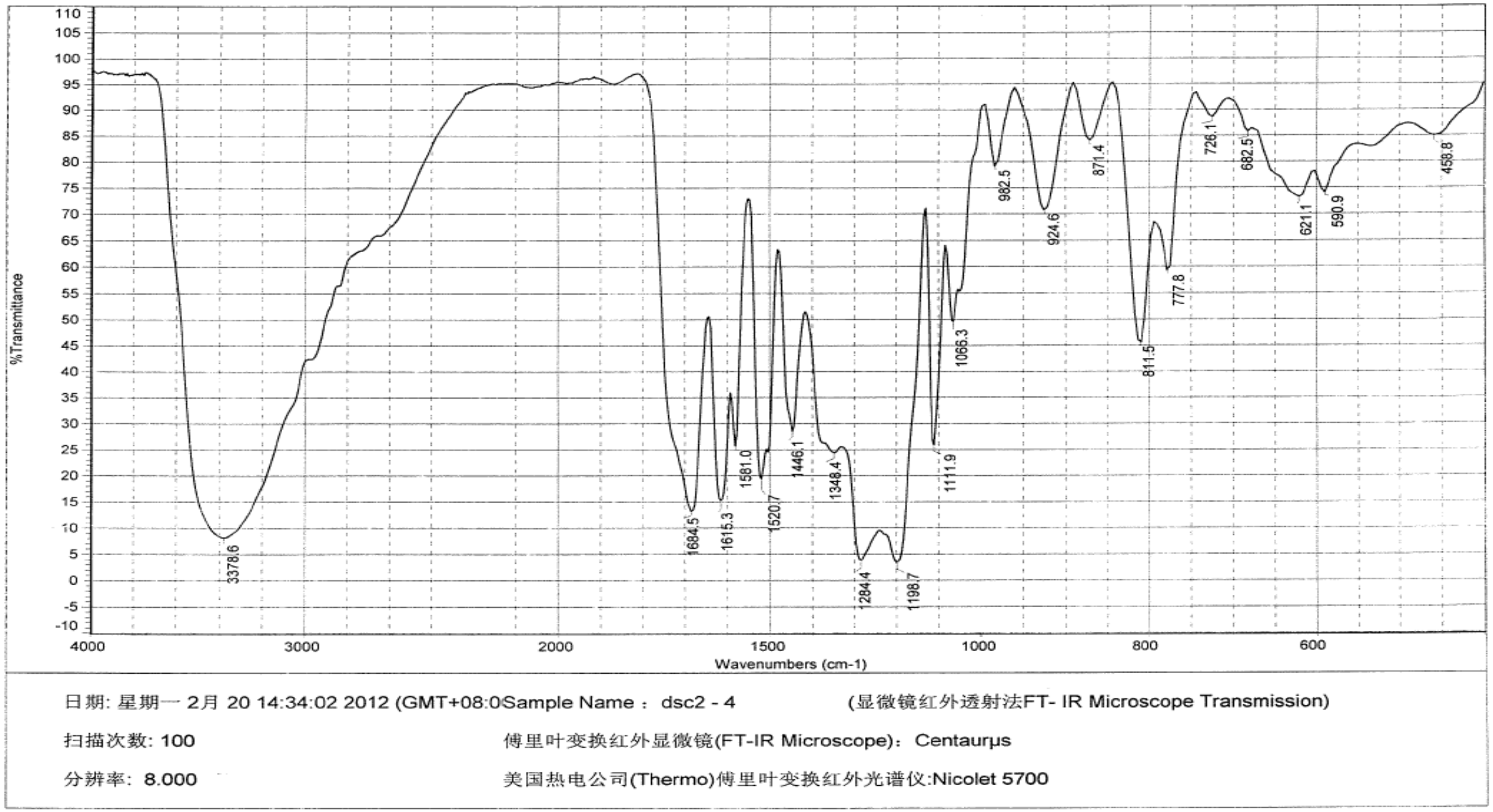

Date: Monday, Feb 20th, 14:34:02 2012 (GMT+08:0Sample Name: dsc 2-4) (Microscopy infrared transmission method FT-IR Microscope Transmission)

Numbers of scan: 100 Fourier transform infrared spectroscopy (FT-IR Microscope): Centaurus

Resolution ratio: 8,000 America Thermo Electron Corporation (Thermo) Fourier transform infrared spectrometer: Nicolet 5700

Figure S15. ^{1}H NMR spectrum (acetone-d_6, 500 MHz) of compound **2**.

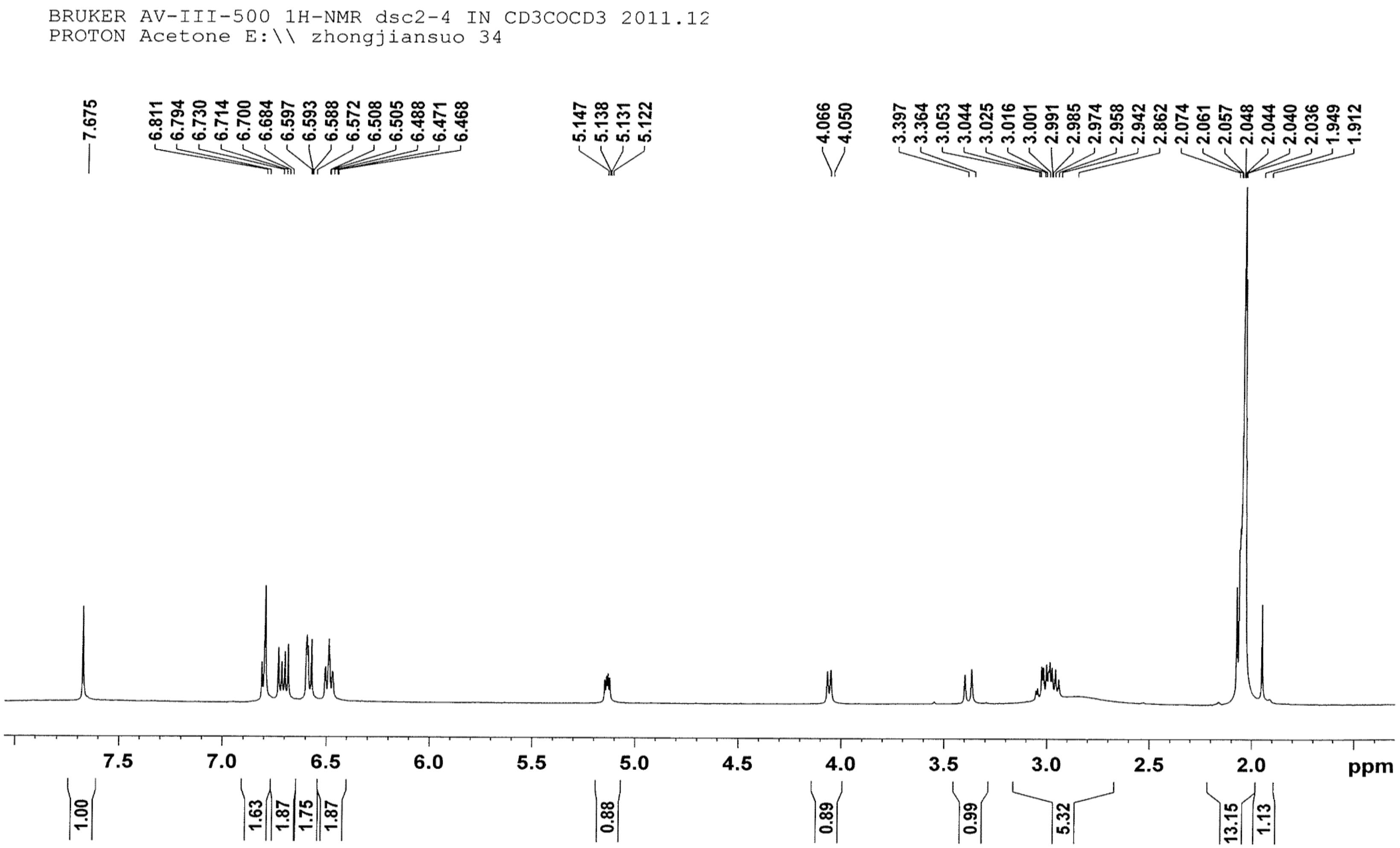

Figure S16. ^{13}C NMR spectrum (acetone-d_6, 125 MHz) of compound **2**.

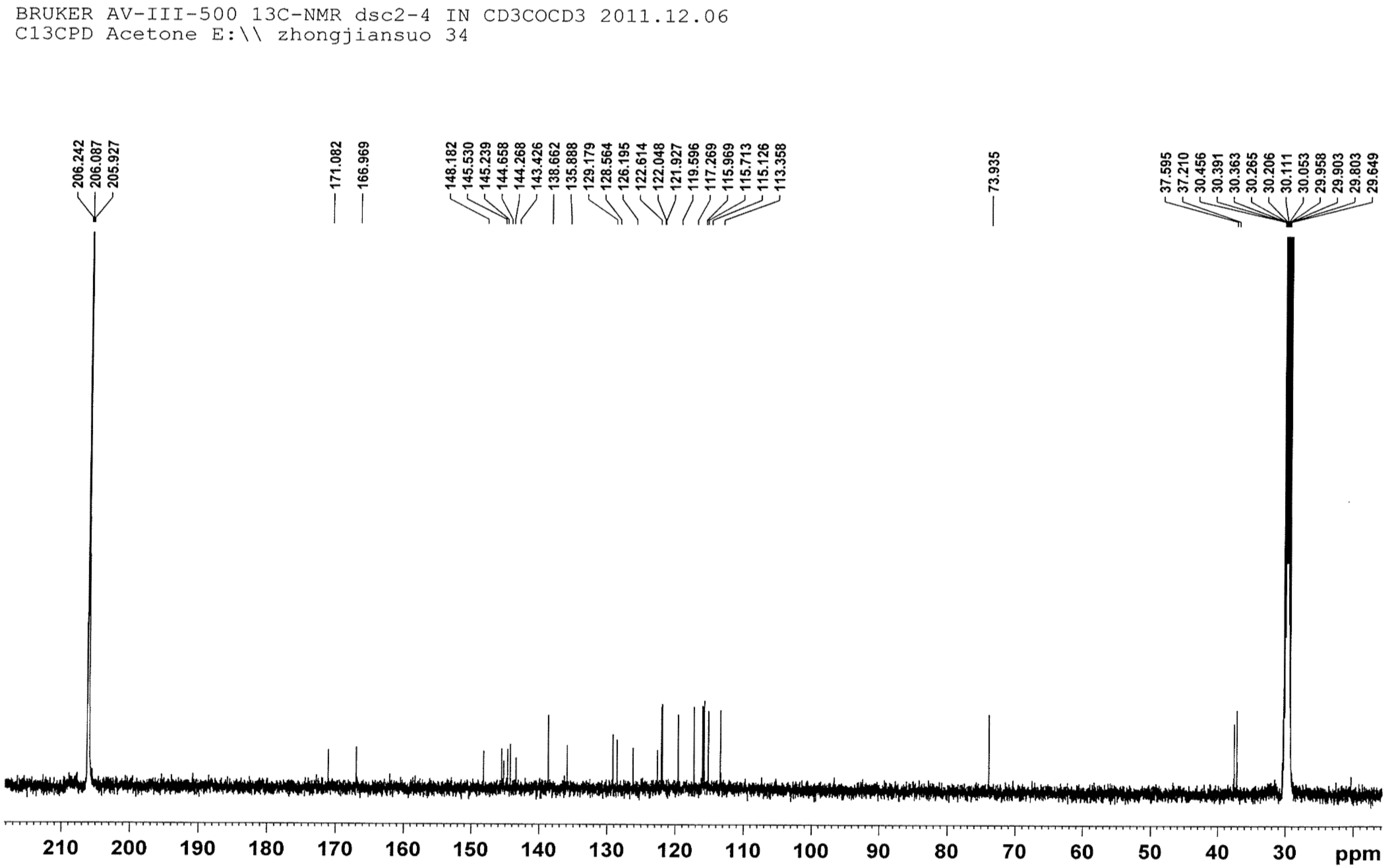

Figure S17. DEPT spectrum of compound **2.**

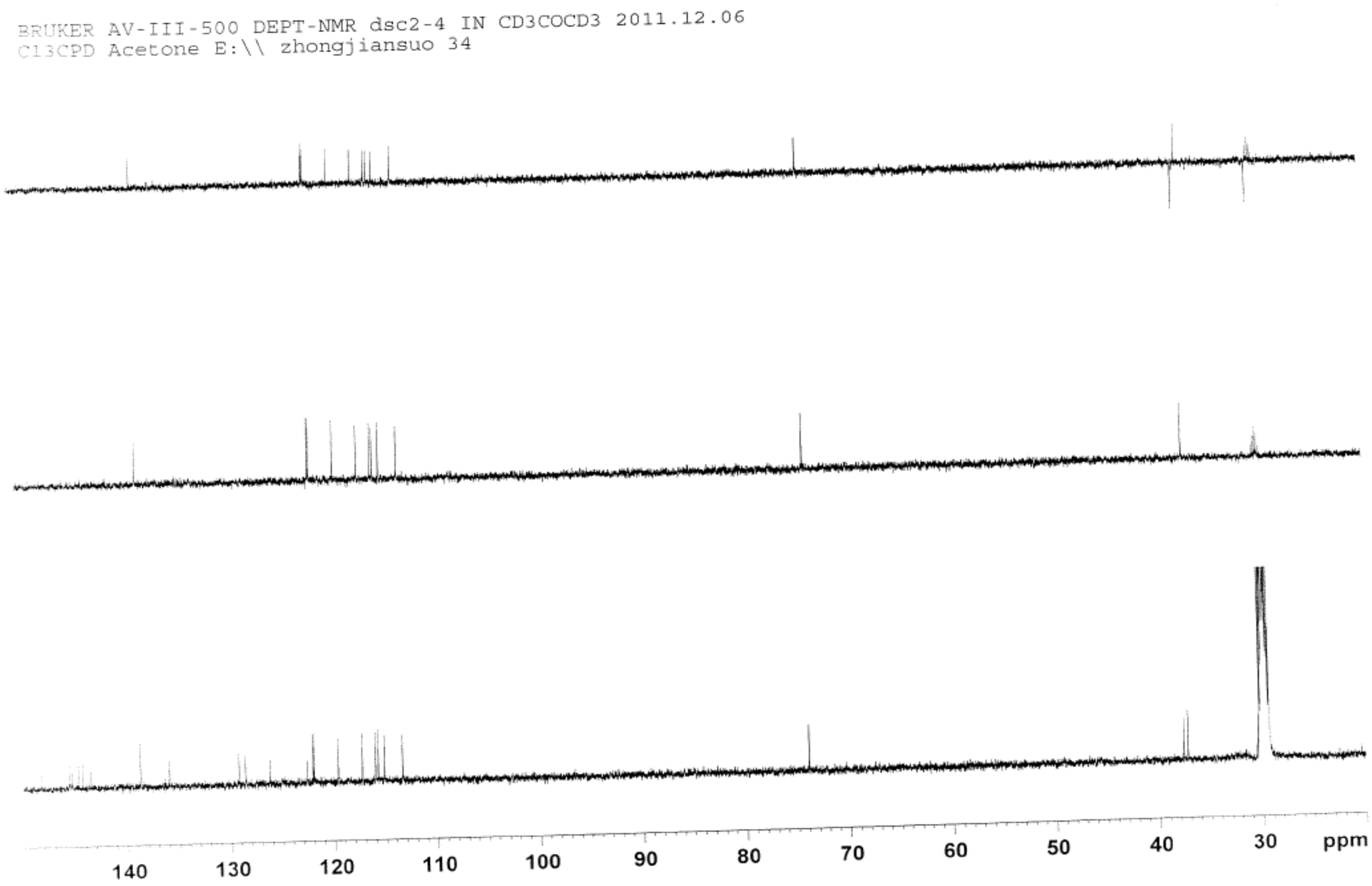

Figure S18. HSQC spectrum of compound **2**.

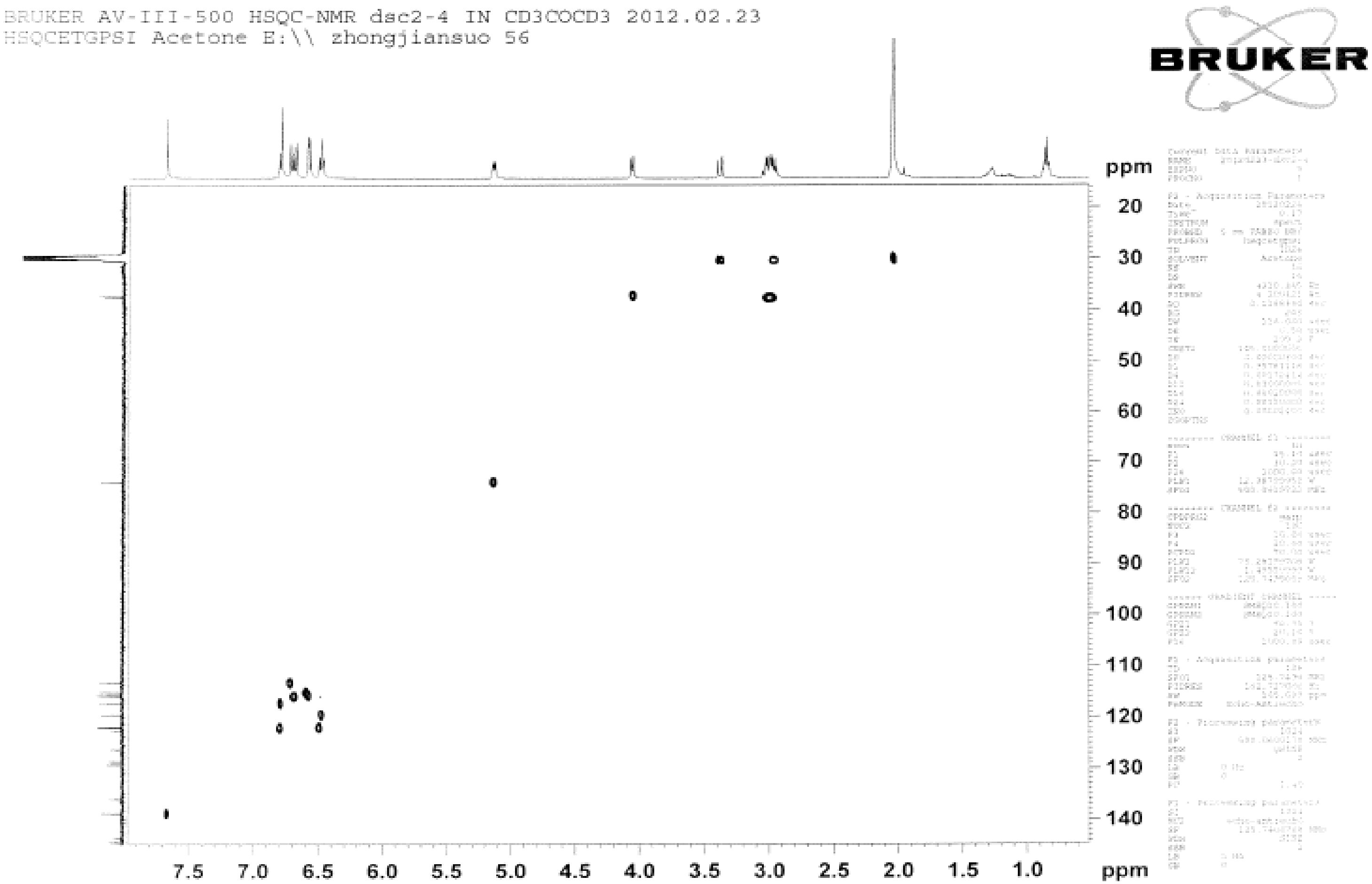

Figure S19. HMBC spectrum of compound **2**.

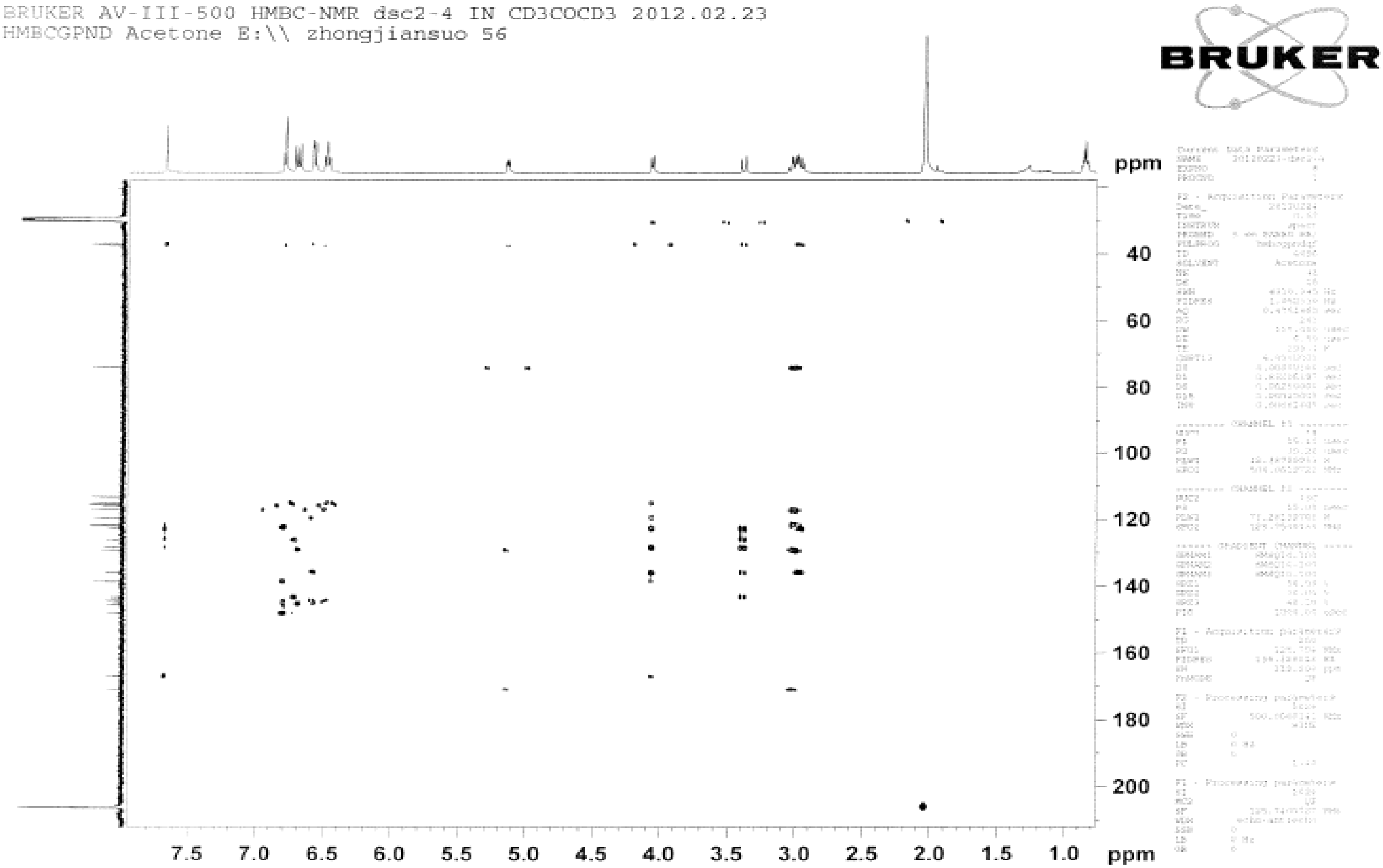

Figure S20. ^{1}H-^{1}H COSY spectrum of compound **2**.

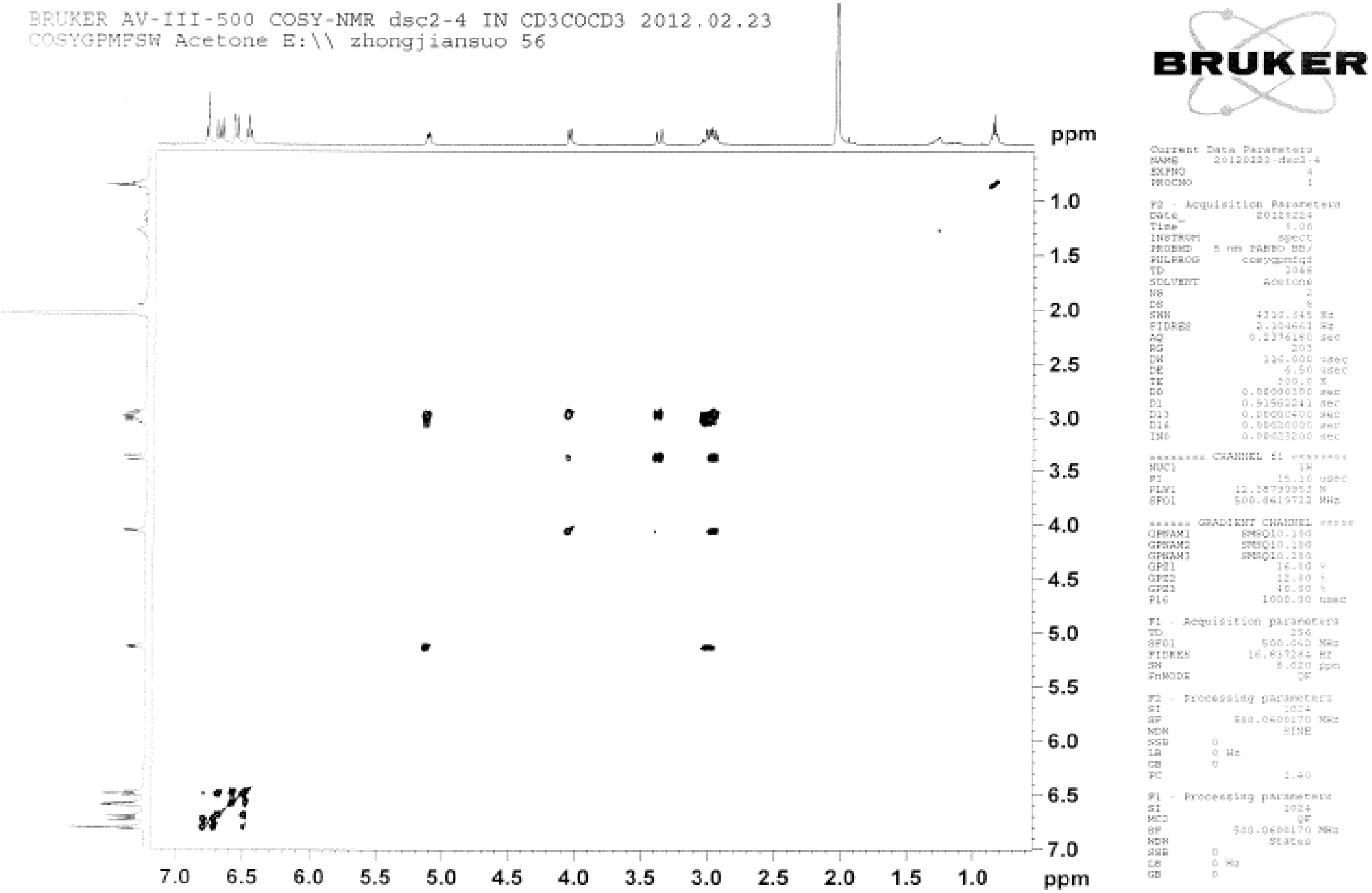

Figure S21. HPLC-UV-ESI-MS^n Chromatograms of Danshen Injection (lyophilized powder).

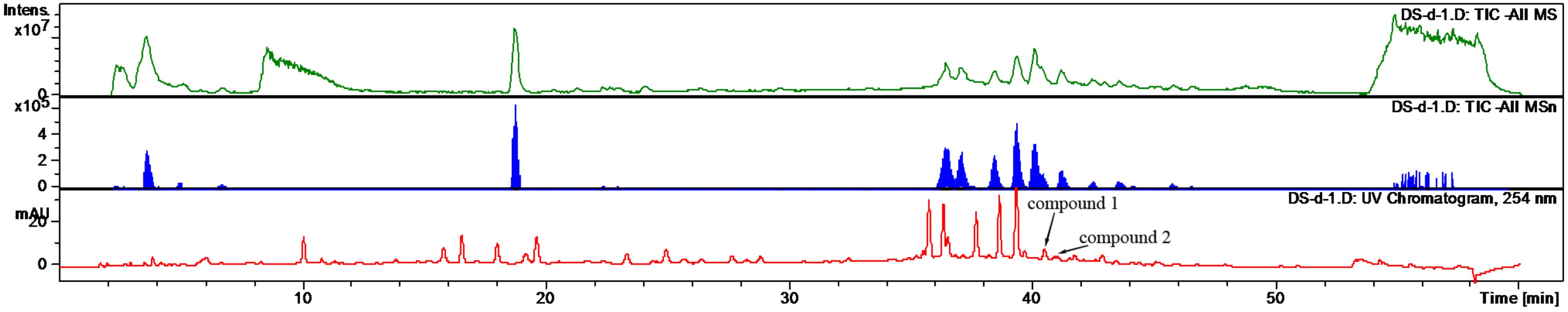

TIC-MS, TIC-MS^n and HPLC chromatograms (from top to bottom) (HPLC: Compound **1**, t_R = 40.5 min; Compound **2**, t_R = 41.1 min).

Apparatus and Chromatographic condition

HPLC: Agilent 6320 LC/MSD mass spectrometer; Column: Germany MACHEREY-NAGEL NUCLEODUR C_{18} Pyramid (4.6 mm × 250 mm, 5 μm); Mobile phase: gradient elution by 0.1% formic acid (HCOOH)-acetonitrile (CH_3CN), see table below; Wavelength: 254 nm; Flow: 1.0 mL min^{-1} (Split ratio, 4:1); Column temperature: 30 ℃; Concentration: 0.2 mg/mL; Injection volume: 5 μL.

Time (min)	0	8	30	45	50	51	55	56	60
A. 0.1%HCOOH (%)	100	97	80	55	55	10	10	100	100
B. CH_3CN (%)	0	3	20	45	45	90	90	0	0

MS: Nebulizer: 30.0 psi; Gas flow: 9.0 L/min; Temperature of drying gas: 350 ℃; Capillary voltage: 3500 V; Ionization mode: negative ESI; scan: *m/z* 100–1000.
